# Effect of Housing Types on Growth, Feeding, Physical Activity, and Anxiety-Like Behavior in Male Sprague-Dawley Rats

**DOI:** 10.3389/fnut.2016.00004

**Published:** 2016-02-04

**Authors:** Jennifer A. Teske, Claudio Esteban Perez-Leighton, Emily E. Noble, Chuanfeng Wang, Charles J. Billington, Catherine M. Kotz

**Affiliations:** ^1^Department of Nutritional Sciences, University of Arizona, Tucson, AZ, USA; ^2^Minneapolis VA Health Care System, Minneapolis, MN, USA; ^3^Minnesota Obesity Center, University of Minnesota, Saint Paul, MN, USA; ^4^Department of Food Science and Nutrition, University of Minnesota, Saint Paul, MN, USA; ^5^Center for Integrative Medicine and Innovative Science, Universidad Andres Bello, Santiago, Chile; ^6^Department of Integrative Biology and Physiology, University of California Los Angeles, Los Angeles, CA, USA; ^7^Department of Medicine, University of Minnesota, Minneapolis, MN, USA; ^8^Geriatric Research Education and Clinical Center, Minneapolis VA Health Care System, Minneapolis, MN, USA

**Keywords:** environmental enrichment, stress, microenvironment, cognition, anxiety

## Abstract

**Background:**

Animal welfare and accurate data collection are equally important in rodent research. Housing influences study outcomes and can challenge studies that monitor feeding, so housing choice needs to be evidence-based. The goal of these studies was to (1) compare established measures of well-being between rodents housed in wire grid-bottom floors with a resting platform compared to solid-bottom floors with bedding and (2) determine whether presence of a chewable device (Nylabone) affects orexin-A-induced hyperphagia.

**Methods:**

Rodents were crossed over to the alternate housing twice after 2-week periods. Time required to complete food intake measurements was recorded as an indicator of feasibility. Food intake stimulated by orexin-A was compared with and without the Nylabone. Blood corticosterone and hypothalamic BDNF were assessed.

**Results:**

Housing had no effect on growth, energy expenditure, corticosterone, hypothalamic BDNF, behavior, and anxiety measures. Food intake was disrupted after housing cross-over. Time required to complete food intake measurements was significantly higher for solid-bottom bedded cages. The Nylabone had no effect on orexin-A-stimulated feeding.

**Conclusion:**

Well-being is not significantly different between rodents housed on grid-bottom floors and those in solid-bottom-bedded cages based on overall growth and feeding but alternating between housing confounds measures of feeding.

## Introduction

Well-being of laboratory animals is a concern of the scientific community and affects validity of scientific data (1, 2), but definitions and methods to measure well-being in rodents varies (3, 4). The microenvironment, including the primary enclosure or cage type, temperature, humidity, illumination, ventilation, and air quality affects physiology, behavior, and disease susceptibility (2). Systematic investigation is needed to determine the independent effect of housing, in-house transfer, and cage modification on behavior, indices of growth, and neuromodulators that influence energy balance (5).

Routine monitoring of objective measures of health and well-being, including growth and behavior, is recommended to ensure that the basic needs of the animal are met (3, 6–9). Historically, rodents have been housed in wire-bottom cages for purposes of sanitation (2), and this housing has also been used to obtain accurate food intake data because spillage can be collected without disturbing animal behavior (10). The 2011 revision of the *Guide for the Care and Use of Laboratory Animals (i.e., The Guide)* states that “flooring should be solid, perforated, or slated with a slip-resistant surface” and “recognizes that individual circumstances might justify an alternative strategy” (2). This is in contrast to the 1996 *Guide* that recommended solid-bottom housing with bedding for rodents (1, 2). The different recommendations for floors between editions likely reflect varying opinions regarding the well-being results across studies.

In some limited instances, wire-bottom grid cages have been reported to promote foot lesions and prevent rodents from performing species-specific behaviors, such as gnawing, nest building, and foraging (10). However, well-being as indicated by body weight and food intake has not been reported to be statistically different between animals housed in wire and solid-bottom cages (11–19). Preference of rodents for wire or solid-bottom cages is dependent on time of day (12, 20), with wire flooring preferred during the active cycle. Foot lesions have been reported among older rodents housed long-term in wire-bottom cages (8 months to >1 year time on flooring) but absent with short-term housing (10). It has been suggested that rodents consume food particles (e.g., spillage) in solid-bottom cages as evidence of foraging behavior; however, no study has tested whether uneaten food (i.e., spillage) in solid cages differs from spillage that falls beneath wire-bottom cages (12). Gnawing is a species-specific behavior in rodents and provision of a chewing device may promote this behavior, though it is unclear whether rodents consistently use the chewing device or whether the presence of the device affects behavioral outcomes, such as feeding. Frequent cage cleaning with inherent disturbance of rodents has independent effects on study outcomes (21–23) although there has been little testing of behavior (motor, anxiety-like).

The primary goal of the current studies was to compare established measures of well-being, such as feeding behavior, physical activity, anxiety-like behavior, stereotypies, and growth, in addition to determining whether transfer between caging types affected these endpoints. The secondary goal was to determine whether provision of a chewable device, which is commonly provided to rodents for enrichment purposes, influences orexin-A-induced feeding, and the effect of housing on the growth and plasticity factor, brain-derived neurotrophic factor (BDNF).

## Materials and Methods

### Animals

Seven-week-old male Sprague-Dawley rats were purchased from a commercial vendor (Charles River, Kingston, NY, USA). Upon arrival, rats were housed in solid-bottom cages with corn-cob bedding and acclimated to the housing facility for 1 week before study procedures began. Rodents were housed individually to facilitate measurement of food intake from individual rodents with a 12-h light/12-h dark photo cycle (lights on at 0600 hours) in a temperature-controlled room (21–22°C) in a facility accredited by the American Association for Laboratory Animal Science. Harlan Teklad-pelleted chow (8604) and tap water were available *ad libitum*. Where indicated, rodents had *ad libitum* access to a non-nutritive rodent chewing device (Nylabone, Product # K3200, natural flavor, BioServ, Frenchtown, NJ, USA). Animals in study one were housed in stainless steel wire grid-bottom (1-mm-diameter round wires spaced 1 cm apart) or solid-bottom-bedded polycarbonate cages (48.4 cm × 26.7 cm × 20.3 cm). The wire grid-bottom cages (40.6 cm × 24.1 cm × 20.6 cm) contained a resting platform (17.8 cm × 10.5 cm) and were suspended above a collection pan with absorbent paper to facilitate collection of uneaten food particles (e.g., spillage). The solid-bottom cages contained white “Crink-l’Nest” paper bedding (Product # CNW, Andersons Inc., Maumee, OH, USA). Rats in study 2 were housed in wire-bottom cages without resting platforms after arrival from the commercial vendor and throughout the study. Rodents were monitored twice daily for health status. No adverse events or foot lesions were observed during the experimental trials. The studies were approved and carried out in accordance with the recommendations of the Institutional Animal Care and Use Committee at the Minneapolis Veterans Affairs Health Care System and the University of Minnesota.

### Specific Experimental Designs

#### Study 1

**Figure 1:**
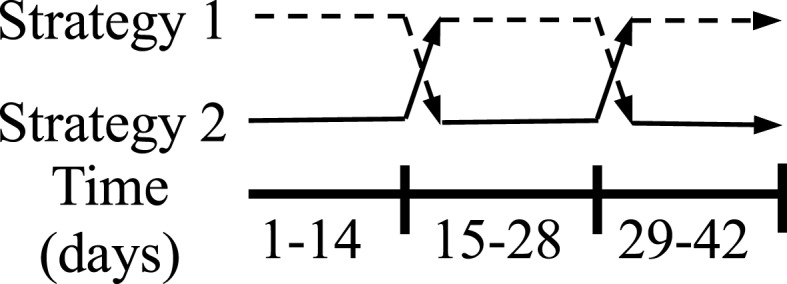
**Experimental design for study 1: rodents in housing type 1 were first housed in wire-bottom cages, then transferred to solid-bottom cages, and finally returned to wire-bottom cages**. Rodents in housing type 2 were first housed in solid-bottom cages, then transferred to wire-bottom cages, and finally returned to solid-bottom cages. Rodents remained in each housing for 2-week periods. *N* = 20 (*n* = 10/housing strategy).

After 1-week acclimation to the housing facility, rodents were stratified by body weight to one of the two housings: (1) wire-bottom + resting platform + Nylabone or (2) solid-bottom-bedded cages with “Crink-l’Nest” bedding + Nylabone (*N* = 20, *n* = 10/initial housing type). Rodents remained in either housing for 2 weeks. Then, rodents in wire cages were moved to solid cages and rodents in solid cages were moved to wire cages. Rodents remained in this new housing for 2 weeks. Finally, rodents were returned to their original housing (Figure 1). Body weight and body composition were measured thrice weekly throughout the three periods. Food intake was defined as the difference between preweighed food and the remaining food less the spillage. Spillage was defined as food particles that fell onto paperboard beneath the wire floor or recovered from bedding in the solid-bottom cage. Collection of food intake measures for rodents in solid-bottom-bedded cages necessitated (1) the removal of rodents from the cage, (2) separation of food and feces particles from paper bedding, and (3) replacing wet or soiled bedding with fresh bedding in standard, measured amounts. Absorbent paperboard beneath rodents housed on wire grid was changed thrice weekly. Rodents were placed in clean solid-bottom cages weekly and rodents in wire-bottom cages were placed in clean cages biweekly based on the standard operating procedures in the facility. Food intake and spillage were recorded 5 days each week. Time required to collect food intake measurements was also measured to determine feasibility of this method for rodent feeding studies.

Anxiety-like behavior was measured by the light/dark test as described below. Spontaneous physical activity (SPA) and energy expenditure were measured concurrently as described previously (24). Rodents were euthanized rapidly by decapitation without anesthesia at 1100. Trunk blood was collected, interscapular brown adipose tissue (iBAT) was dissected out, and the hypothalamic paraventricular nucleus (PVN) was excised with a micropunch technique as described previously (25). Food was removed from rodents 4 h prior to euthanasia (0700) to avoid potential effects of recent food intake on endpoint measurements. Corticosterone was measured in plasma by radioimmunoassay with a commercial kit (Product #07120102, MP Biomedicals, Solon, OH, USA). Uncoupling protein 1 (UCP1) mRNA in iBAT was measured by RT-PCR previously (26). BDNF protein levels in the PVN were measured with a commercial ELISA kit (Product #KA0330, Abnova, Taipei, Taiwan) as described previously (27).

#### Study 2: Effect of the Presence of a Nylabone on Feeding Stimulated by Orexin-A

After completing experiment 1, a group of rodents (*n* = 13) were maintained in wire-bottom cages and surgically prepared with a single cannula directed toward the rostral lateral hypothalamus as described previously (28). Orexin-A (500 pmol/0.5 μL) or vehicle-control (artificial cerebrospinal fluid) was injected into the rostral lateral hypothalamus through the cannula in a latin-square counter-balanced design as described previously (29). Rats were randomized to one of four treatment groups (orexin-A with Nylabone, orexin-A without Nylabone, vehicle-control with Nylabone, and vehicle-control without Nylabone). Each animal received each treatment once, and all treatments were represented on a given day. Food intake was measured 1 and 2 h postinjection. Food intake was determined as the difference between preweighed food minus the food weight at 1 and 2 h postinjection and spillage.

### Body Composition Measurement

Total fat and fat-free mass were measured using a quantitative magnetic resonance body composition analyzer (EchoMRI-900™, Houston, TX, USA) as described previously (30, 31). Percent fat and fat-free mass were calculated as the ratio of absolute fat or fat-free mass to body weight, respectively.

### Indirect Calorimetry and SPA Measurement

Energy expenditure and SPA were measured in a solid-bottom customized eight-chamber continuous open-circuit indirect calorimeter/SPA system after day 42 as described previously (24). The system was designed to measure simultaneous and continuous O_2_ consumption, CO_2_ production (Columbus Instruments, Columbus, OH, USA) (32), and SPA (Product #MED-OFA-RS, Med Associates, St. Albans, VT, USA) in each chamber. Gas sensors were calibrated prior to each test with a primary gas standard. Chamber air-flow was maintained at 3.1 L/min and experiments were performed at 22°C. Rodents were acclimated to the chamber for 3 days prior to the 24-h test and food and water were available *ad libitum* during acclimation and test periods. VO_2_ and VCO_2_ were measured at 30-s intervals and reference measurements from room air were determined at 15-min intervals over the 24-h testing period. From O_2_ consumption and CO_2_ production measurements, total energy expenditure over the 24-h measurement period was calculated as the sum of heat measurements during the 24-h period. Resting energy expenditure was defined as the lowest metabolic rate during the light cycle extrapolated over 24-h. From the SPA measurements, time spent ambulating (locomotor activity), time spent in rearing, and time spent grooming (performing stereotypies) were calculated by the Med Associates Software. Stereotypies were defined as movement within a 4-cm radius around the rodent.

### Light/Dark Test

Anxiety-like behavior was measured by the light/dark test as described previously (33) with minor modifications. Briefly, a dark box insert (Product #ENV-516, Med Associates, St. Albans, VT, USA) was placed into SPA chambers (43.2 cm × 43.2 cm, Med Associates, St. Albans, VT, USA). The dark box insert created an equal sized illuminated and dark compartment (21.6 cm × 42.5 cm) with an opening (11.4 cm × 8.9 cm) to allow movement between the compartments. Naïve rats were placed in the light compartment facing away from the dark compartment to initiate the 15-min test. The number of entries (into the light compartment, into the dark compartment, and total) was assessed by two methods concurrently (manually and automatically). Entries were manually scored by a trained observer and automatically defined by the photocells of the SPA chambers and the Med Associates computer software. Manually scored entries were considered valid if two paws (both forelimbs) or if all four paws (forelimbs and hindlimbs) entered into the compartment. The software determined time spent in each compartment. At the end of the 15-min test, rats were returned to their home cage. The SPA chambers and dark box were cleaned with 70% ethanol between tests.

### Cannulation Surgery

Animals were anesthetized with ketamine (50 mg/kg) and xylazine (15 mg/kg), and a 26-gauge stainless steel cannula (Product #C315G/SPC, Plastics One, Roanoke, VA, USA) was directed toward the rostral lateral hypothalamus as described previously (28, 29, 34, 35). Stereotaxic coordinates (2.2 mm posterior and 1.9 mm lateral to bregma, and 7.3 mm below the skull surface) were determined from the rat brain atlas of Paxinos and Watson (36). Experimental trials began 7–10 days after surgery. Postsurgical analgesia was administered on the day of surgery and for 2 days after surgery [flunixin meglumine (Banamine), 2.5 mg/kg, i.p., Merck Animal Health, Madison, NJ, USA].

### Drugs

Orexin-A (Product #003-30, Phoenix pharmaceuticals, Burlingame, CA, USA) was dissolved in artificial cerebrospinal fluid and artificial cerebrospinal fluid was used as the vehicle-control. Drugs were stored at 4°C for <48 h prior to injection.

### Injections

Injections were performed as described previously (28, 29, 34, 35, 37). Briefly, 0.5 μL of orexin-A or artificial cerebrospinal fluid was injected over 30 s with a 33-gauge injector (Product #C315I/SPC, Plastics One, Roanoke, VA, USA) that extended 1.0 mm beyond the tip of the guide cannula. The injector was left in place for an additional 10 s. Extensive tissue damage is absent after 50 repeated injections, as measured by gliosis around the injection site and light microscopy at 100× (38). Injections were given to naïve rats at 1300 hours in the home cage and at least 48 h elapsed between treatments to allow for drug clearance.

### Gene Expression and Protein Analysis

The PVN and iBAT tissues were frozen immediately in liquid nitrogen following excision and were stored at −80°C until analysis. Relative UCP1 and glyceraldehyde 3-phosphate dehydrogenase gene expression in the iBAT was measured by one-step real-time RT-PCR. Relative UCP1 mRNA is expressed as a ratio of UCP1 to the housekeeping gene, glyceraldehyde 3-phosphate dehydrogenase, as described previously (26). BDNF in the PVN was measured with a commercially available ELISA kit and was expressed as a ratio of BDNF to total protein as described previously (27).

### Total RNA Extraction and Quantification

Total RNA was isolated using the Qiagen RNeasy Plus micro kit (Product #74034, Qiagen, Valencia, CA, USA) with minor modifications previously (28). Briefly, tissue was homogenized with Trizol reagent (Product #T9424 Sigma-Aldrich, St. Louis, MO, USA) and chloroform. After phase separation, the aqueous phase was removed and applied to a gDNA column. Total RNA was precipitated with 70% ethanol and applied to a MiniElute column. The concentration and purity of the total RNA were determined by the 260- and 280-nm readings on a spectrophotometer (Nanodrop ND-1000, Nanodrop Technologies, Wilmington, DE, USA).

### One-Step Real-Time RT-PCR

Primers for UCP1 and glyceraldehyde 3-phosphate dehydrogenase were created using MacVector 7.2 (Accerlys, San Diego, CA, USA). One-step real-time RT-PCR was performed using 100 ng of total RNA and the Roche RNA Amplification Kit SYBR Green I (Roche Applied Science, Product #12015137001, Indianapolis, IN, USA) previously (28). Primer annealing and data acquisition for glyceraldehyde 3-phosphate dehydrogenase and UCP1 was completed at 58 or 61°C (annealing) and was 82 or 84°C (data acquisition), respectively.

### Brain-Derived Neurotrophic Factor ELISA

Protein extraction was performed using a method by Baker-Herman et al. with minor modifications (39) as described previously (27). Briefly, brain punches were homogenized in cold extraction buffer (Boston BioProducts, Product #BP-119, Ashland, MA, USA) with HALT protease inhibitor cocktail (Thermo Scientific, Product # 78420B, Rockford, IL, USA). Homogenates were acidified, incubated at room temperature, and neutralized. Homogenates were centrifuged and supernatants collected. Protein was quantified from supernatant using a Bradford assay. BDNF quantification was performed using a BDNF sandwich ELISA kit (Abnova, Product #KA0330m, Taipei City, Taiwan), according to the manufacturer’s instructions. Samples were diluted with sample dilution buffer, such that 30 μg of protein were added to each well. Plates were read at 450 nm. Samples were assayed and analyzed in duplicate.

### Plasma Corticosterone

Trunk blood was collected following decapitation in EDTA tubes and placed on ice. Rodents were brought into the euthanasia room just prior to decapitation without anesthesia to mitigate stress. Blood was centrifuged (2000 × *g*) for 30 min, and plasma was separated and stored at −80°C until analysis. Plasma corticosterone was measured with a commercially available radioimmunoassay kit (MP Biochemicals, Product #07120102, Solon, OH, USA) according to the manufacturer’s instructions. Samples and standards were run in duplicate or triplicate, respectively, and measured on a gamma counter. This assay is highly specific, cross-reacting at <1% with other hormones.

### Statistical Analyses

#### Software and General Aspects

For all statistical tests, *N* = 10/group in study 1 and *N* = 13 for study 2. Based on the historic weight gain data, 10 rats per group were needed to detect a significant difference with 80% power, an effect size = 1.35, and alpha = 0.05. For all statistical tests, *n* = 10/group in study 1 and *N* = 13 for study 2. The unit of analysis was a single animal. Statistical analyses were performed using R software version 3.1.2 (40) or Prism 5.0 (GraphPad Software, Inc., San Diego, CA, USA). Linear mixed models were fitted with the *lme4* package (41) using R software. Diagnostics plot for mixed models were tested for normality of random effects and residual distribution. For linear mixed models, statistical significance for fixed effects was analyzed with the likelihood ratio test using the command ANOVA (R base). All code and data are available from the authors upon request. All data are expressed as mean ± SEM. *P* values <0.05 were considered significant.

#### Analysis of Study 1

To analyze the effect of housing in solid vs. wire on body weight, fat, and fat-free mass change over time, we used a linear mixed model approach. For each endpoint, we initially fitted a model including fixed effects for cage type (solid vs. wire), sequence of cage switch (solid–wire–solid and wire–solid–wire), time-point of measurement nested within period (corresponding to each stage of the study), and an interaction between cage and period. The interaction between cage and period was not significant, and thus, the final model included only main effects for the fixed effects. We included random intercepts and slopes for rats to account for within- and between-rat variation over time. Statistical significance for fixed effects was evaluated by a likelihood ratio test of the full model against the model lacking the factor in question.

**Figure 2:**
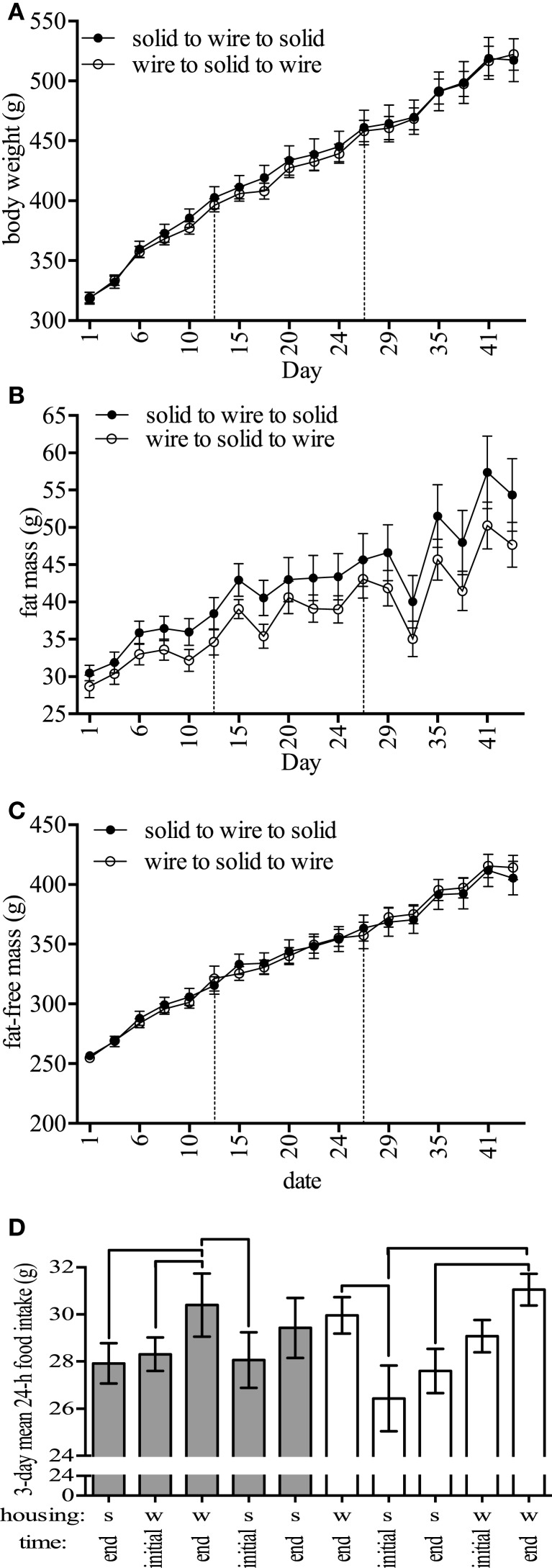
**Body composition and feeding-related outcomes**. **(A)** Body weight, **(B)** fat mass, **(C)** fat-free mass, and **(D)** 24-h food intake during 3-day periods at the beginning and end of 2-week periods in rodents housed in either solid (s)- or wire (w)-bottom cages for 2 weeks (days 1–14), moved to the other housing for 2 weeks (days 15–28), and then moved back to the original housing for 2 weeks (days 29–42). For food intake **(D)**, note the decrease in food intake after switching from wire to solid cages. Data represent mean ± SEM. Please note different *y*-axes. *N* = 20 (*n* = 10/housing strategy). Lines above bars in **(D)** are significantly different.

To study the effect of cage type on food intake, we used two different analyses. First, we used a linear mixed model to analyze how cage type affected food intake over time. In this model, we included fixed effects for cage type (solid vs. wire), sequence of cage switch (solid–wire–solid and wire–solid–wire), time point of measurement nested within period (corresponding to three measurements at the beginning and end of each period), and an interaction between cage and period. We included random intercepts and slopes for each individual rat to account for within- and between-rat variation over time. The interaction between cage and period was not significant, and thus, the final model included only main effects for the fixed effects. The second analysis focused on the effect of cage switch on food intake. For this analysis, the dependent variable was change in food intake around 3 days of switching caging conditions and was analyzed using a linear mixed model with fixed effects for cage switch type (solid-to-bottom or bottom-to-solid) and fat-free mass as covariate and random intercept for each rat. For representation purposes, we conducted pairwise *t*-tests within each caging sequence, which were corrected by multiple comparisons to illustrate changes in food intake over time (Figure 2D).

In addition to the above analyses for study 1, we tested for differences between cage types for anxiety-like behavior, metabolism, brown fat uncoupling protein, and BDNF with *t*-tests adjusted with the Bonferroni correction (GraphPad Software). Thus, a *P*-value <0.0033 was considered to be statistically significant for these 15 *t*-tests [i.e., *P* = 0.0033 (0.05/15)].

#### Analysis of Study 2

The effect of the Nylabone on orexin-A-induced feeding was analyzed with two factor repeated measures ANOVA where there were two within-subject factors (Graphpad 5.0). “Dose” (orexin-A or control injection) and “Nylabone” (presence or absence) were the repeated measures. There was no main effect of the Nylabone on food intake and the “dose” × “Nylabone” interaction was not significant; therefore, two *post hoc* comparisons were completed with the Sidak test, which corrected for multiple comparisons. This allowed determination of whether there was a difference in response between animals given vehicle and orexin-A in the presence and absence of the Nylabone separately.

## Results

### Study 1

#### Growth

Upon randomization to initial housing, body weight was not statistically different between housing (*t*-test, solid- and wire-bottom: 245.0 ± 2.8 and 244.3 ± 3.4 g, *P* = 0.87). A linear mixed model showed that cage type affected body weight [χ^2^(1) = 7.34, *P* = 0.006] and fat-free mass [χ^2^(1) = 3.97, *P* = 0.046] change over time (Figures 2A,C). Estimated effects from the model indicated that housing in a wire cage compared to a solid-bottom cage increased body weight by 2.08 ± 0.76 g and fat-free mass by 1.33 ± 0.67 g, corresponding to 1.0 and 0.9% of the overall change in each parameter for the standard housing condition. We found no significant effect of cage type on fat mass change over time [χ^2^(1) = 0.42, *P* = 0.51, Figure 2B]. In this analysis, there were no significant effects of cage sequence on body weight [χ^2^(1) = 1.11, *P* = 0.29], fat [χ^2^(1) = 0.79, *P* = 0.37], and fat-free mass change [χ^2^(1) = 1.89, *P* = 0.16].

#### Food Intake

Estimated effects from a linear mixed model analysis indicated that housing in a wire cage compared to a solid-bottom cage increased food intake by 1.86 ± 0.01 g [χ^2^(1) = 16.11, *P* = 0.00005, Table 1], which represented 0.14% of the overall change in food intake. We found no significant effect of cage sequence [χ^2^(1) = 0.32, *P* = 0.57]. To understand whether these effects were influenced by the order of the cage switch, we tested whether cage switch (solid-to-bottom or bottom-to-solid) affected acute food intake by comparing food intake between the last 3 days of housing and the first and last 3 days of measurement in a new housing condition (Figure 2D). These analyses indicated that switching from a wire to a solid-bottom cage decreased food intake during the first 3 days by 4.15 ± 0.08 g [χ^2^(1) = 19.74, *P* = 0.000008]. In Figure 2D, we show the food intake for each stage, indicating pairwise comparisons within each cage sequence to illustrate changes in food intake over time for each cage sequence. Finally, we tested for an effect of cage type and cage change sequence on food spillage. We found no significant effect of cage [χ^2^(1) = 1.21, *P* = 0.27] or cage sequence [χ^2^(1) = 0.49, *P* = 0.48] on food spillage (data not shown). The amount of time required to complete food intake measurements was greater in solid-bottom cages compared to wire-bottom cages (solid- and wire-bottom: 253.2 ± 4.8 and 61.6 ± 1.5 s, respectively, data not shown). Together, these data suggest that animals housed in wire cages have a small, but statistically significant increase in food intake, and that switching from solid to wire cage causes an acute increase in food intake.

**Table 1:** **Body composition, total food intake, and spillage**.

	Days		
	1–14	15–28	29–42
**Body weight gain (g)**			
Solid-bottom	92.4 ± 7.0	54.8 ± 4.4	54.4 ± 2.8
Wire-bottom	87.4 ± 6.0	53.1 ± 7.1	56.0 ± 4.8
**Fat mass gain (g)**			
Solid-bottom	12.5 ± 1.5	2.8 ± 1.2	10.8 ± 1.4
Wire-bottom	10.4 ± 1.2	3.6 ± 1.9	8.4 ± 1.2
**Fat-free mass gain (g)**			
Solid-bottom	76.4 ± 5.7	47.1 ± 3.6	43.3 ± 2.5
Wire-bottom	70.7 ± 4.8	35.2 ± 4.8	43.1 ± 4.0
**Total food intake (g)**			
Solid-bottom	396 ± 11.3	405.3 ± 9.0	423.8 ± 18.0
Wire-bottom	407.3 ± 10.0	428.3 ± 16.6	439.9 ± 9.3
**Total spill (g)**			
Solid-bottom	26.1 ± 7.1	22.7 ± 3.3	26.8 ± 2.3
Wire-bottom	19.9 ± 2.7	26.1 ± 2.8	22.8 ± 2.6

*Data represent mean ± SEM. *N* = 20 (*n* = 10/housing strategy)*.

#### Behavior

Anxiety-like behavior was not significantly different between animals housed in different conditions according to the results of the light–dark box test. There were no differences in time spent in the light and dark compartments (Table 2) or entries in either compartment independent of whether the entries were observer defined (solid- and wire-bottom: 15.2 ± 2.1 entries and 12.9 ± 2.5 entries) or computer defined (solid- and wire-bottom: 50.3 ± 5.2 entries and 55.0 ± 10.4 entries). Similarly, there were no differences in anxiety-like behavior as measured in the SPA test. Stereotypies, indicated by time spent grooming, were not statistically different between housing (solid- and wire-bottom: 46.8 ± 4.8 and 38.2 ± 3.2 min, respectively). Time spent ambulating (48.4 ± 3.7 and 47.0 ± 7.2 min) and rearing (26.2 ± 2.9 and 22.3 ± 3.1 min) were not statistically different between solid- and wire-bottom cages, respectively.

**Table 2:** **Time spent in the light and dark compartment during the light–dark box test in rats housed in solid- or wire-bottom housing**.

	Light compartment		Dark compartment	
	Solid-bottom	Wire-bottom	Solid-bottom	Wire-bottom
Time moving (min)	8.2 ± 1.1	8.7 ± 1.2	6.8 ± 1.1	6.2 ± 1.2
Time ambulatory (min)	4.5 ± 0.6	3.9 ± 0.5	3.2 ± 0.4	2.8 ± 0.4
Time vertical (min)	2.3 ± 0.4	2.2 ± 0.3	0.9 ± 0.1	1.0 ± 0.2

*Data represent mean ± SEM. *N* = 20 (*n* = 10/housing strategy)*.

#### Metabolism, Brown Fat Uncoupling, Brain-Derived Neurotrophic Factor

**Figure 3:**
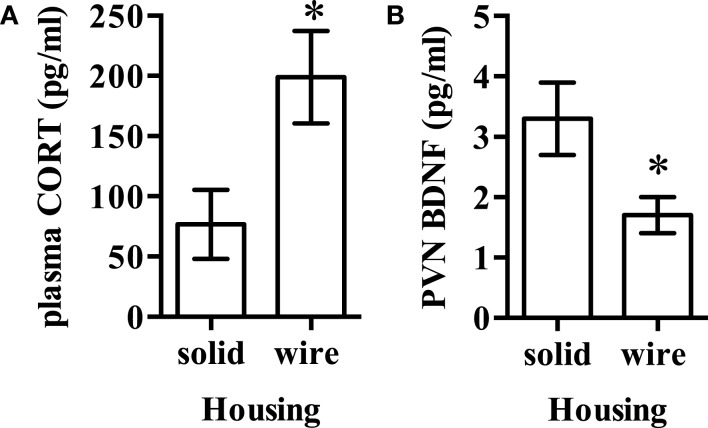
**(A)** Plasma corticosterone and **(B)** brain-derived neurotrophic factor (BDNF) in the hypothalamic paraventricular nucleus (PVN) was not significantly different after Bonferroni correction for multiple comparisons between rodents housed in either solid- or wire-bottom cages. Data represent mean ± SEM. Please note different *y*-axes. *N* = 20 (*n* = 10/housing strategy). *P < 0.05 as compared to solid-bottom housing.

Total (60.9 ± 1.8 and 59.4 ± 1.6 kcal) and resting energy expenditure (43.4 ± 1.4 and 41.8 ± 1.5 kcal) were not statistically different between animals housed in solid- and wire-bottom housing (*P* > 0.05 for all comparisons). Uncoupling protein 1 mRNA in iBAT was not significantly different in rodents housed in solid-bottom cages (solid- and wire-bottom: 46.9 ± 4.8 and 36.7 ± 2.8, *P* = 0.08). Plasma corticosterone (solid- and wire-bottom: 76.8 ± 28.5 and 199.0 ± 38.4 ng/mL, *P* < 0.02) was not significantly greater in rodents housed in wire-bottom cages while PVN BDNF [solid- and wire-bottom (pg/μg of total protein): 3.3 ± 0.6 and 1.7 ± 0.3, *P* < 0.02] was not significantly greater in rodents housed in solid-bottom cages after Bonferroni correction for multiple comparisons (Figure 3).

### Study 2: Effect of a Chewing Device, Nylabone, on Cumulative Short-Term Feeding Stimulated by Orexin-A

**Figure 4:**
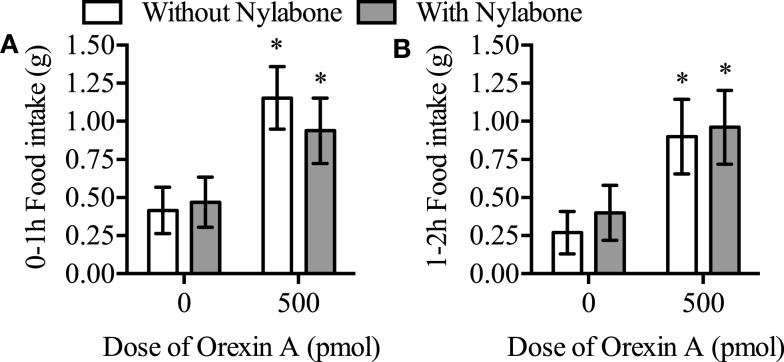
**Orexin-A injection into the rostral lateral hypothalamus stimulated feeding in the presence and absence of a Nylabone during the (A) 0-1 and (B) 1-2 h postinjection time periods in Sprague-Dawley rats housed on wire-bottom floors**. *N* = 13. Data represent mean ± SEM. *P < 0.05 as compared to vehicle-control both in the presence and absence of the Nylabone.

There was no main effect of the Nylabone on food intake and the interaction between presence of the Nylabone and orexin-A treatment was not significant during the 0–1 and 1–2-h postinjection feeding periods (*P* > 0.05, Figure 4). In contrast, there was a main effect of orexin-A treatment on food intake 0–1 and 1–2 h postinjection [*F*_(1,12)_ = 21.0, *P* = 0.0006 and *F*_(1,12)_ = 5.1, *P* = 0.0428, respectively]. Orexin-A significantly increased food intake in the presence and absence of the Nylabone 0–1 (*P* = 0.0064 and *P* = 0.0002, respectively, Figure 4A) and 1–2 h postinjection (*P* = 0.0063 and *P* = 0.0028, Figure 4B).

## Discussion

Modifications to policies regarding rodent housing must be balanced with the feasibility of conducting scientific studies (42) because routine husbandry (cage change, single vs. group housing, provision of shelters, and food placement) affects energy balance-related endpoints, such as sleep and weight gain (5, 43). Empirical studies are needed because changes in rodent-housing policies have independent effects on study outcomes. We determined the effect of two different housing types on established measures of well-being, behavior, metabolic markers, physiological measures, and indicators of study feasibility. We also tested whether the presence of a Nylabone influenced feeding stimulated by centrally administered orexin-A. The data show that the following endpoints were either minimally different or not statistically different between the tested housing strategies: body weight and composition, food intake, feed efficiency, spillage, behavior, energy expenditure, and brown fat UCP1 gene expression. In contrast, transferring rats to different housing situations had a significant effect on food intake immediately after the move. Time required to complete food intake measurements was greater in solid-bottom caging. After correcting for multiple comparisons, PVN BDNF expression and plasma corticosterone were not different between animals housed on different types of flooring (wire vs. solid). Presence of a Nylabone did not affect feeding stimulated by orexin-A. Together these data suggest that (1) housing rodents in wire-bottom caging with a resting platform does not reduce well-being, (2) in-house transfer between cage types affects feeding, and (3) providing a Nylabone, a chewing device, does not affect orexin-A-stimulated food intake.

Our analysis with linear mixed model estimated that housing in wire compared to that in solid-bottom increased weight gain by 2.08 g and fat-free mass by 1.33 g, which is less than the SEM for both weight gain and fat-free mass gain. Despite that this result was statistically significant, these estimates represent 1.0 and 0.9% of the overall gain in weight and fat mass, respectively (Table 1). Thus, over this 6-week period, our data are consistent with studies that have found housing did not affect absolute body weight (11–13), weight gain (14), and motor activity after an acclimation period (12, 14–16). Others have reported that absolute body weight or weight gain was greater (13, 17) or less (18, 19) among rats housed on wire-bottom floors. Greater physical activity during the active period has been noted among rats housed on wire-bottoms (14). Discrepancies between our study and others are likely due to the differences in methodology related to rodent preferences for cage type (12, 20), bedding material (44), age (18), or dietary protein composition (13). Preference studies show that rodents spend more time on solid surfaces during the light cycle (resting/sleeping period) and wire-surfaces during the dark cycle (active period) (12, 20) and prefer fibrous bedding material (44). Thus, we included a resting platform in our wire-bottom cages and used paper contact bedding in our solid-bottom cages to accommodate rodent preferences (42). This may have contributed to the lack of differences observed in our study and may explain why our study contrasts with others (13, 14, 17–19).

Consistent with others (14, 17, 45, 46), we found no effect of housing on total energy expenditure, resting energy expenditure, brown fat UCP mRNA, an indicator of thermal sensitivity (47), or physical activity, which contributes to total energy expenditure. Some have thought that wire caging and lack of bedding pose a thermal stress for rats (46), but we found no evidence for that. Both the acute (24-h total and resting energy expenditure) and chronic (brown fat) measures of energy expenditure were not statistically different between housing conditions, suggesting that rodents housed in our wire grid floor cages were not thermally stressed. The longer term indicator of thermal sensitivity, brown fat UCP1, was determined after rats had been housed in their respective housing for a duration sufficient to detect changes in brown fat UCP1 (48). Lack of thermal stress may be related to ambient temperatures in the animal colony.

Discrepancies in results for physical activity measures between our study and others may be due to differences in acclimation time in the testing chambers (34) and the time-dependent preferences for cage floor mentioned previously. We had expected differences in physical activity between groups, as contact bedding has been suggested to increase physical activity in the home cage environment (17). Further, moving rats from wire- to a solid-bottom cage has been shown to reduce physical activity in the home cage environment (49). It is plausible that physical activity as measured in the current study – a non-home-cage-testing environment – either did not reflect home-cage activity or that the finding in the testing environment was influenced by the housing environment. For instance, SPA may have been dampened among wire-housed rodents, since the SPA measurement chambers have a solid-bottom floor; this could then mask potential differences between groups.

Our analysis with linear mixed model estimated that housing in wire compared to solid-bottom cages increased food intake by 1.86 ± 0.01 g, which is less than the SEM for food intake (Table 1). While this result was statistically significant, that amount of food intake represented only 0.14% of the total food intake. This would not have a large physiological impact, and thus, we concluded that housing did not affect total food intake. In contrast, one study reported greater food intake in rodents housed on wire (12), while another showed that food intake was gender and strain dependent (50). It has been suggested that differences in food intake between housing paradigms may be due, in part, to the inability of rodents housed in wire to forage and “recover” food that fell beneath the wire floor (12, 49). If this were the case, then spillage in solid-bottom caging would have been less than in wire-bottom cages, but spillage was not significantly different between cage types.

Transferring rats between housing significantly affected food intake and food intake measurement from solid-bottom cages took significantly more time. Thus, our results extend prior reports showing that rats reduce food consumption after being transferred to a clean cage, even without altering the housing type (23), routine rodent husbandry affects study outcomes important to energy balance research (5, 51) and study feasibility (42). Food intake measurements require precise determination of spillage and measurements must be completed in a timely manner to ensure between-subject consistency (52). The time required to complete food intake measurements in solid-bottom bedded cages was 3–5 times longer than in wire-bottom cages and food intake was significantly reduced after rodents were moved to the other housing. These data align with others showing that cage changes have significant effects on measurements of food intake and thus can affect physiological measures (5). Less frequent cage changes are associated with fewer defensive behaviors (21), lower anxiety-like behavior (53), and altered physiological parameters (49, 54–60).

We tested whether a chewing device influenced short-term feeding since modifications to housing practices have been shown to affect feeding (43). Food intake stimulated by orexin-A (28, 29, 35) was not statistically different in the presence and absence of the Nylabone chew device. This suggests that provision of this chewing device following habituation does not influence this behavioral outcome. Additionally, we can conclude from these data that the hyperphagic effect of orexin-A is not secondary to mastication, since the Nylabone allows for that behavior *ad libitum*. These results do not address whether the Nylabone is a salient object for rodents, if a chewing device influences other behavioral outcomes or if other modifications to the rodent’s environment affects behaviors relevant to energy balance research.

We determined if housing affects anxiety-like behavior with the light/dark box test and stereotypic movement. The light/dark box test is a validated method to assess anxiety-like behavior, whereby an increased number of crossings between light and dark compartments and increased time spent in the light compartment indicates increased anxiety-like behavior (33). Grooming or stereotypic movement is sensitive to changes in acute and chronic stress (61). Excessive stereotypies are abnormal behaviors associated with suboptimal aspects of the environment and thus may indicate reduced well-being (6, 8). Housing did not affect time spent in, or entries into, the light or dark compartments during the light/dark box test. This is consistent with other reports (62, 63) showing no effect of housing type (referred to as “impoverished, standard or enriched”) in the elevated plus maze test, light/dark box test, tail-suspension test, forced-swim test, and social interaction task. Stereotypic movement was not statistically different across cage types in our study, which is consistent with another study (12). Thus, these behavioral tests for anxiety-like behavior, stress, and well-being indicate no difference between housing.

In agreement with the behavioral tests, plasma corticosterone was not significantly different between housing when corrected for multiple comparisons. This is consistent with some (11, 64, 65) and contrasts others who showed rats housed on wire-bottom floors had more (16) or less (17) corticosterone (from urine, plasma, or feces) compared to rats housed in solid-bottom bedded caging. The reason for these inconsistencies is not readily apparent but parallels the literature on the inconsistent affects of environmental enrichment (66, 67) and husbandry (68) on corticosterone and highly variable corticosterone measurements (4–64% variability between rodents) among animals in the same housing condition (6). Changes in glucocorticoid levels are associated with exposure to stressful circumstances [e.g., electric shock and social defeat (4, 61)]. Although corticosterone is commonly used to indicate existence of stress, glucocorticoids as “universal indicators of stress or measures of poor welfare” is problematic (69–73), and it is unclear whether changes in glucocorticoids parallel changes in welfare (6). Based on our data and others, modifications to housing have inconsistent effects on behavioral and physiological measures of anxiety, stress, and well-being, and a single measure is inappropriate for assessing the effect of housing on well-being.

We determined the effect of housing on BDNF in the hypothalamic PVN, which affects energy metabolism (74). Hypothalamic BDNF is elevated by environmental enrichment (75), and BDNF in the PVN reduces food intake (76) and increase energy expenditure (32). Furthermore, BDNF in the PVN has been shown to directly regulate corticotropin-releasing hormone production (77), and corticotrophin-releasing hormone receptor signaling pathways have been implicated to mediate the affects of BDNF on energy balance (78). We found no significant effect of cage type on BDNF in the PVN after multiple comparisons correction. We did not observe differences in energy balance or food intake, suggesting a contribution of additional systems to our measured behavioral and physiological effects. Thus, it is plausible that an independent effect of housing on BDNF may prevent comparison between studies utilizing different housing.

We acknowledge limitations of our study that prevent generalizability to all rodent studies. First, as shown previously, cage change frequency may have had an independent effect on food intake (23) and other endpoints in this study. Second, energy expenditure by indirect calorimetry, as is customary with most calorimetric systems, was measured outside the home-cage after a 3-day acclimation period; as such, energy expenditure measurements may reflect this new environment instead of the respective home cage setting. Third, despite that rodents housed on wire-bottom floors had resting platforms and Nylabones, lack of bedding on wire-bottom floors could have had an independent effect on the endpoints. Finally, handling was not balanced between the two cage types. Future studies would be needed to test the independent effects of cage change frequency, home-cage energy expenditure, and bedding/nesting material on feeding behavior and on indices of well-being.

In conclusion, these data indicate that well-being was not different between rodents in housing with these specific caging systems (wire-bottom with resting platform and Nylabone vs. solid-bottom bedded cages with “Crink-l’Nest” bedding + Nylabone) based on multiple objective measures of growth; behavior, including motor, stereotypic, and anxiety-like; and energy expenditure. Levels of plasma corticosterone and BDNF were not statistically different between housing types after correcting for multiple comparisons. These data support less frequent husbandry procedures, including transferring rodents between housing types, since this process significantly changed feeding behavior. In addition, these data improve scientific understanding of cage change procedures in energy balance studies and highlight the need to balance animal research concerns with animal well-being such that neither one is neglected. Together, these data indicate the need to either standardize housing practices to reduce confounding effects of the microenvironment on the validity, reliability, and repeatability of scientific behavior studies, and at minimum, to encourage reporting of housing practices to facilitate data interpretation.

## Author Contributions

All authors listed, have made substantial, direct and intellectual contribution to the work, and approved it for publication.

## Conflict of Interest Statement

The authors declare that the research was conducted in the absence of any commercial or financial relationships that could be construed as a potential conflict of interest. The reviewer Andrew Brown and handling Editor Daniel Larry Smith Jr. declared their shared affiliation, and the handling Editor states that the process nevertheless met the standards of a fair and objective review.
